# Machine Learning-Based Radiomics Predicts Radiotherapeutic Response in Patients With Acromegaly

**DOI:** 10.3389/fendo.2019.00588

**Published:** 2019-08-27

**Authors:** Yanghua Fan, Shenzhong Jiang, Min Hua, Shanshan Feng, Ming Feng, Renzhi Wang

**Affiliations:** ^1^Department of Neurosurgery, Peking Union Medical College Hospital, Chinese Academy of Medical Sciences and Peking Union Medical College, Beijing, China; ^2^School of Electrical Engineering and Automation, East China Jiaotong University, Nanchang, China

**Keywords:** acromegaly, radiomics, radiotherapeutic response, magnetic resonance imaging, receiver operating characteristics

## Abstract

**Background:** Prediction of radiotherapeutic response before radiotherapy could help determine individual treatment strategies for patients with acromegaly.

**Objective:** To develop and validate a machine-learning-based multiparametric MRI radiomics model to non-invasively predict radiotherapeutic response in patients with acromegaly.

**Methods:** This retrospective study included 57 acromegaly patients who underwent postoperative radiotherapy between January 2008 and January 2016. Manual lesion segmentation and radiomics analysis were performed on each pituitary adenoma, and 1561 radiomics features were extracted from each sequence. A radiomics signature was built with a support vector machine using leave-one-out cross-validation for feature selection. Multivariable logistic regression analysis was used to select appropriate clinicopathological features to construct a clinical model, which was then combined with the radiomics signature to construct a radiomics model. The performance of this radiomic model was assessed using receiver operating characteristics (ROC) analysis and its calibration, discriminating ability, clinical usefulness.

**Results:** At 3-years after radiotherapy, 25 patients had achieved remission and 32 patients had not. The clinical model incorporating seven clinical features had an area under the ROC (AUC) of 0.86 for predicting radiotherapeutic response, and performed better than any single clinical feature. The radiomics signature constructed with six radiomics features had a significantly higher AUC of 0.92. The radiomics model showed good discrimination abilities and calibration, with an AUC of 0.96. Decision curve analysis confirmed the clinical utility of the radiomics model.

**Conclusion:** Using pre-radiotherapy clinical and MRI data, we developed a radiomics model with favorable performance for individualized non-invasive prediction of radiotherapeutic response, which may help in identifying acromegaly patients who are likely to benefit from radiotherapy.

## Introduction

Acromegaly is a rare chronic disease caused by pituitary somatotroph adenoma that causes high growth hormone (GH)/insulin-like growth factor 1 (IGF1) levels and reduced life expectancy (1, 2). The aim of treatment is to normalize the level of IGF1, which usually reflects appropriate disease control, reduces the risk of complications, and reduces mortality (3–5). The consensus and guidelines for acromegaly recommend transsphenoidal surgery as the first-line treatment, followed by medicinal treatment, with radiotherapy as the third-line choice (6, 7).

The initial cure rate of transsphenoidal surgery for acromegaly patients with macroadenomas is 40–50% when the surgery is performed by experienced pituitary surgeons (7). In comparison with surgery, the drugs used for medicinal treatment, such as somatostatin agonists and pegvisomant have higher costs, require lifelong injections, may be subject to tolerance problems, and may also be ineffective in some drug-resistant adenomas (8). Radiotherapy as a third-line treatment is mainly aimed at patients whose GH and IGF1 levels are poorly controlled, or whose tumors continue to grow after surgery or medication (7, 9). Also, radiotherapy can also be an option for patients who cannot or unwilling to undergo surgery (10). Previous studies have shown that radiotherapy can increase the remission rate for patients with acromegaly who have not been cured through surgery (8, 11, 12). However, radiotherapy still has some problems that cannot be ignored. Radiotherapy can lead to long-term hypopituitarism, visual damage, cranial nerve defects, and increased risk of developing a second brain tumor, cognitive dysfunction, or cerebrovascular disease (7, 9, 13, 14). Therefore, we should focus on the effectiveness of radiotherapy at controlling GH/IGF-1 hypersecretion and identify those patients who would benefit most from radiotherapy, and find a balance between its merits and shortfalls (14).

Thus, an effective pre-radiotherapy prediction method that enables the precise prediction of radiosensitivity and radiotherapeutic response is important in the selection of treatment options and the formulation of individual treatment strategies, and could help avoid the possible side effects and economic burden of radiotherapy for patients with a poor radiotherapeutic response. However, to the best of our knowledge, no previous study has investigated a pre-radiotherapy prediction model for determining radiotherapeutic response in patients with acromegaly.

Radiomics is an emerging machine learning method that can extract numerical data reflecting biologically important tissue characteristics from medical imaging information (15). Compared with traditional methods, data mining in radiomics has two unique advantages. First, the radiomics method allows the semi-automatic or automatic extraction of radiomics features and offers abundant data relative to qualitative analyses. Second, high-dimensional radiomics information can shed light on the heterogeneity within a region through identifying different sub-regions and defining the spatial complexity of disease (16). Various recent studies have demonstrated that radiomics is a particularly promising approach for assisting in developing individual treatment strategies in oncology (17, 18). We therefore hypothesized that radiosensitivity in patients with acromegaly may be related to high-dimensional information present in MRI images, and developed an MRI-based radiomics model to predict radiotherapeutic response in patients with acromegaly.

## Methods

### Patients

Acromegaly patients admitted to the Department of Neurosurgery of Peking Union Medical College Hospital (PUMCH) between January 2008 and January 2016 were enrolled in this study. The diagnostic criteria for acromegaly were: (1) adult patients with clinical features of acromegaly (7); (2) a pituitary adenoma detected by pituitary magnetic resonance imaging (MRI); (3) meeting the endocrine diagnostic criteria for acromegaly (7) (elevated IGF-1 levels (19), random GH level >1 ng/mL, and nadir GH level > 0.4 ng/mL after OGTT).

The inclusion criteria were: (1) patients with a history of stereotactic radiosurgery (SRS) or fractionated stereotactic radiotherapy (FSRT); (2) a pituitary contrast-enhanced MRI examination before radiotherapy, and pituitary adenomas with a maximum diameter of not <8 mm can be detected; (3) complete pre-radiotherapy clinical data (as shown in clinical data collection before radiotherapy); and (4) follow-up of more than 3-years after radiotherapy.

A complete pituitary hormonal evaluation was performed before radiotherapy. Patients who had undergone operation or medical treatment after radiotherapy were excluded. After collection and screening, 57 acromegaly patients were identified for this retrospective study, which was approved by the ethical review committee of the Peking Union Medical College Hospital, and the patients' informed consent was obtained.

### Clinical Data Collection Before Radiotherapy

The pre-radiotherapy clinical features collected included gender, age, random GH value, IGF-1 standard deviation score (SDS), nadir GH value, and GH inhibition ratio after OGTT, tumor volume, Knosp grade (20), tumor consistency (21) (soft or firm), cavernous sinus invasion (collected from the surgical video), Ki-67 value (<3 or ≥3), and P53 value (negative or positive). A calculator available online (http://ticemed_sa.upmc.fr/sd_score/) was used to obtain individual IGF-I SDS after entering the age, gender, IGF-I value and assay name (22).

### Management After Radiotherapy

FSRT combines stereotactic localization with fractionated therapy, with small doses being administered over several sessions. The FSRT protocol used in this study involved administering a total dose of 50 Gy in 28 or 25 sessions (5 sessions per week) (23). All patients who underwent SRS were treated with gamma knife, which delivers adequate radiation doses (14–34 Gy) to the tumor in one session (23).

Complete hormonal, clinical, and pituitary MRI examinations were performed at 3 and 6 months after radiotherapy, and annually thereafter in our out-patient service. The efficacy of radiotherapy and the radiotherapeutic response were determined by hormone levels at 3-years after radiotherapy. According to the latest guidelines, remission was defined by (1) either basal GH <1.0 ng/mL or nadir GH <0.4 ng/mL after OGTT; and (2) a normal age- and gender- adjusted IGF-1 level (6, 7) without additional drug therapy. Patients who failed to meet the above criteria were considered as non-remission. The same GH or IGF1 test was used for an individual patient during the follow-up period.

### Tumor MRI Imaging Segmentation and Radiomics Feature Extraction

All patients underwent pituitary MR enhanced imaging using a 3.0-T magnetic resonance device (Discovery MR 750, GE Healthcare, Chicago, IL, USA). The pre-radiotherapy pituitary MRI protocol consisted of three sequences: T1-weighted imaging (T1WI), contrast-enhanced T1WI (CE-T1), and T2-weighted imaging (T2WI). A neuro-radiologist with 7 years of experience delineated regions of interest (ROIs) representing the tumor on all MRI images using ITK-SNAP software (University of Pennsylvania, www.itksnap.org). Then, all ROIs were manually checked by a neuro-radiologist with 12 years of experience without any prior knowledge of the patients. All differences were settled through negotiation between the two readers.

Then, the open-source PyRadiomics package (24) (https://github.com/Radiomics/pyradiomics) was used to extract radiomics features from the ROIs, and all features were normalized to a value between 0 and 1. Four types of features were calculated, including (1) first order features (*n* = 180), (2) textual features [*n* = 680, including gray-level co-occurrence matrix (GLCM), gray-level run-length matrix (GLRLM) and gray-level size-zone matrix (GLSZM)], (3) wavelet features (*n* = 688), and (4) shape and size features (*n* = 13).

### Radiomics Feature Selection

The feature selection process can improve the performance of an algorithm by reducing the over-fitting caused by redundant and irrelevant information in high-dimensional data. In this study, the support vector machine (SVM) method was used to predict whether a patient would achieve remission. A radiomics score was calculated for each patient using an SVM model with a linear kernel based on the selected features. Values of C ϵ [0.01, 1] with a step size of 0.01 were tested. Feature selection was conducted using a leave-one-out cross-validation (LOOCV) procedure (25), with the LOOCV being used to determine the optimal value of the regularization parameter C. In each iteration of the LOOCV, the patients were divided into two groups, with 56 patients (N – 1, with N was the patient number of our study) being used for training and only one for testing, with the process being repeated 57 times. The C value maximizing the average prediction accuracy (ACC) was selected as the optimal regularization parameter, and then the most important radiomics features were identified using the selected C value.

### Radiomics Analysis

The radiomics signature was constructed using the selected radiomics features and an SVM with a radial basis function (RBF) kernel. The clinical features used to develop the clinical prediction model were selected using multivariable logistic regression analysis with selection according to the Akaike information criterion (AIC) (26). Finally, a nomogram (radiomics model) (27) combining the radiomics signature and clinical model was constructed using multivariate logistic regression.

Receiver operating characteristic (ROC) (28) curve analysis was performed, and the values of the area under the ROC curve (AUC), ACC, sensitivity (SN), specificity (SP), positive predictive value (PPV), and negative predictive value (NPV) were also used to demonstrate predictive performance. The DeLong test was used to compare the predictive efficacy of the three models.

Calibration curves and the Hosmer-Lemeshow test were used to evaluate the similarity between the predicted and observed radiotherapeutic response probabilities (29). Decision curve analysis (DCA) was performed to assess the clinical usefulness of the radiomics model by quantifying the net benefits at different probability thresholds (30).

### Statistical Analysis

A two-sided *p*-value < 0.05 was considered to be statistically significant. All statistical analyses were performed by a dedicated statistician using SPSS software version 22 (SPSS Inc., Chicago, USA) and R statistical software version 3.4.1 (R Foundation for Statistical Computing, Vienna, Austria). The tumor volume was accessed through 3D slicer (31) (version 4.10.2, http://www.slicer.org). The calibration plot was analyzed using the “hdnom” package, and DCA was analyzed using the function “dca.R.”

## Results

### Clinical Characteristics

**Figure 1:**
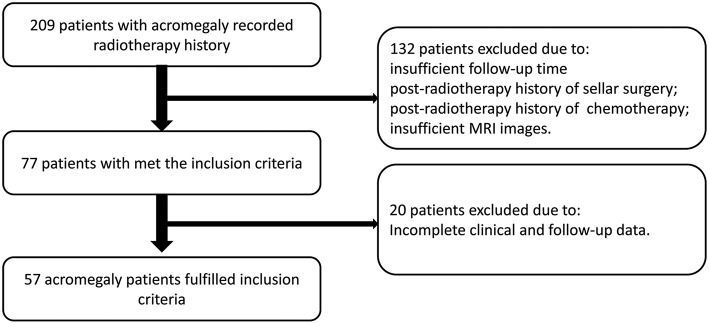
Patient recruitment pathway.

Since 2008, a total of 209 patients with acromegaly recorded radiotherapy history at our hospital. The FSRT was performed at PUMCH, and gamma knife procedures were performed in other hospitals. After screening, 132 patients were excluded because of one or more of the following: insufficient follow-up time, post-radiotherapy history of sellar surgery or chemotherapy, or insufficient MRI images. Twenty patients were excluded because of incomplete clinical data. Finally, 57 patients with acromegaly met the screening criteria and were included in this study (Figure 1), 34 patients underwent FSRT treatment, while 23 patients underwent gamma knife treatment. And the histopathological type of all included patients is GH secreting adenoma.

The clinical characteristics of these 57 patients are summarized in Table 1. In the 3-year follow-up after radiotherapy (according to the latest consensus and remission criteria for acromegaly patients), 25 patients (FSRT: 14 and gamma knife: 11) achieved remission and 32 patients did not. There were no significant difference in radiotherapeutic response between the FSRT and Gamma Knife groups (*P* = 0.620).

**Table 1:** Patients' characteristics.

**Characteristic**	**Whole Set** **(*n =* 57)**	**Remission** **(*n =* 25)**	**Non-remission** **(*n =* 32)**	***P*-value**
**Gender**				
Male	20 (35.1%)	9 (36.0%)	11 (31.4%)	0.898
Female	37 (64.9%)	16 (64.0%)	21 (68.6%)	
**Age (year)**	38.62 ± 12.78	40.89 ± 15.04	36.84 ± 10.60	0.239
**GH level (ng/mL)**	5.30(3.15-11.60)	3.8(2.7-5.8)	9.1(4.35-17.96)	0.000
**Nadir GH level (ng/mL)**	3.38(2.06-8.41)	2.11(1.65-3.61)	6.55(2.35-13.83)	0.000
**IGF-1 SDS**	4.43 ± 1.59	4.24 ± 1.49	4.58 ± 1.70	0.276
**GH inhibition ratio (%)**	35.05 ± 18.28	41.24 ± 21.56	30.21 ± 13.74	0.022
**Tumor volume (mm3)**	2.04(0.94–5.65)	1.28(0.407–5.00)	3.00(1.69–6.64)	0.040
**Knosp grade**				
Grade 2	15 (26.3%)	12 (48.0%)	3 (9.4%)	0.004
Grade 3	21 (36.8%)	7 (28.0%)	14 (43.8%)	
Grade 4	21 (36.8%)	6 (24.0%)	15 (46.9%)	
**Tumor consistency**				
Firm	23 (40.4%)	7 (28%)	16 (50.0%)	0.093
Soft	34 (59.6%)	18 (72.0%)	16(50.0%)	
**Cavernous sinus invasion**				
Yes	43 (75.4%)	19 (76.0%)	24 (75.0%)	0.931
No	14 (24.6%)	6 (24.0%)	8 (25.0%)	
**Ki-67(%)**				
<3	35 (61.4%)	17 (68.0%)	18 (56.3%)	0.336
≥3	22 (38.6%)	8 (32.0%)	14 (43.7%)	
**P53**				
Negative	47 (82.5%)	22 (88.0%)	25 (78.1%)	0.331
Positive	10 (15.5%)	3 (12.0%)	7 (21.9%)	

*SDS, standard deviation score. Categorical variables were presented as the number. Continuous variables consistent with a normal distribution were presented as mean ± standard deviation, otherwise the median and quartile are used. Chi-Square or Fisher Exact tests, as appropriate, were used to compare the differences in categorical variables, while the independent sample t-test was used to compare the differences in continuous variables*.

Five pre-radiotherapy clinical characteristics (the random GH value, nadir GH value, GH inhibition ratio after OGTT, tumor volume, and Knosp grade) were significantly associated with the radiotherapeutic response in patients with acromegaly (*P* = 0.000–0.040). Acromegaly patients with low random GH, nadir GH value, tumor volume, Knosp grade, and high GH inhibition ratio value had a better response to radiotherapy and were more likely to achieve remission. No significant differences were found in age, gender, IGF-1 SDS, tumor consistency, cavernous sinus invasion, Ki-67, or P53 value between the different radiotherapeutic response groups.

### Traditional Assessment of the Radiotherapeutic Response

**Figure 2:**
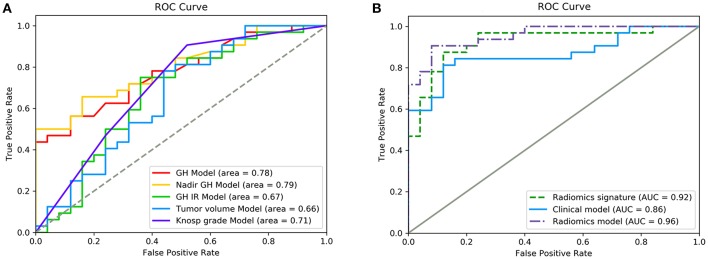
ROC curves showing the performance of different features and models in the prediction of response to radiotherapy. **(A)** Diagnostic performance of each independent clinical feature. **(B)** Diagnostic performance of the radiomics signature, clinical model, and radiomics model. Radiomics features had significantly higher AUC values than the clinical model (*p* = 0.011).

The five significant features mentioned above were used to establish independent logistic regression-based predictive models of the response to radiotherapy (Figure 2A). The AUCs of the ROC curves for random GH, nadir GH, GH inhibition ratio, tumor volume, and for Knosp grade, were 0.78, 0.79, 0.67, 0.66, and 0.71, respectively. The ACC, sensitivity, specificity, PPV, and NPV of the six independent clinical predictive models are shown in Table 2.

**Table 2:** Performance of single clinical features, clinical model, radiomics signature, and radiomic model.

**Model**	**Performance**	**ACC**	**AUC**	**SN**	**SP**	**PPV**	**NPV**
Significant clinical features	GH	0.684	0.78	0.625	0.76	0.770	0.613
	Nadir GH	0.719	0.79	0.656	0.80	0.808	0.645
	GH inhibition ratio	0.649	0.67	0.750	0.52	0.667	0.619
	Tumor volume	0.667	0.66	0.969	0.28	0.633	0.875
	Knosp grade	0.719	0.71	0.906	0.48	0.690	0.8
Clinical model	Combined clinical features	0.807	0.86	0.844	0.76	0.818	0.792
Radiomics signature	Combined radiomics features	0.842	0.92	0.812	0.88	0.897	0.786
Radiomics model	Combined clinical features and radiomics signature	0.912	0.96	0.906	0.920	0.935	0.885

*ACC, accuracy; AUC, area under curve; SN, sensitivity; SP, specificity; PPV, positive predictive value; NPV, negative predictive value*.

Random GH, IGF-1 SDS, GH inhibition ratio, tumor volume, Knosp grade, tumor consistency, and P53 value were selected (according to their AIC values) as discriminatory factors to build the clinical model, which resulted in the AUC value being improved to 0.86 (Figure 2B), with an optimized ACC of 0.807, sensitivity of 0.844, and specificity of 0.76 (Table 2). The clinical model constructed using multivariate logistic regression analysis had better prediction performance than any single clinical feature. And the result indicates that the machine learning method performs better than the conventional method.

### Radiomics Feature Selection and Signature Construction

A total of 1561 quantitative radiomics features could be extracted from each MRI sequence from a single patient. From these, one shape, two textual, and three wavelet features were finally selected using a linear kernel SVM model and a LOOCV procedure. The best regularization parameter (C = 0.14) was determined by LOOCV. Three of the selected features were extracted from T1WI images, two from CE-T1, and one from T2WI images. Further details of the six radiomics features are provided in Table 3.

**Table 3:** The selected six key radiomic features detail information.

**Class**	**Feature name**	**Feature type**	**Stem from**	**Remission**	**Non-remission**	***P*-value**
Exponential	gldm_DependenceVariance	Texture	T1WI	0.385 ± 0.045	0.574 ± 0.036	0.0016
Exponential	glcm_SumEntropy	Texture	T1WI	0.425 ± 0.064	0.190 ± 0.033	0.0011
Wavelet	HHH_firstorder_Kurtosis	Wavelet	T1WI	0.285 ± 0.039	0.437 ± 0.045	0.0171
Wavelet	HHL_gldm_DependenceVariance	Wavelet	CET1	0.514 ± 0.044	0.698 ± 0.027	0.0003
Original	shape_Maximum3DDiameter	Shape and size	CET1	0.513 ± 0.050	0.704 ± 0.027	0.0009
Wavelet	HHH_glszm_SmallAreaLowGrayLevelEmphasis	Wavelet	T2WI	0.355 ± 0.051	0.237 ± 0.034	0.0494

**Figure 3:**
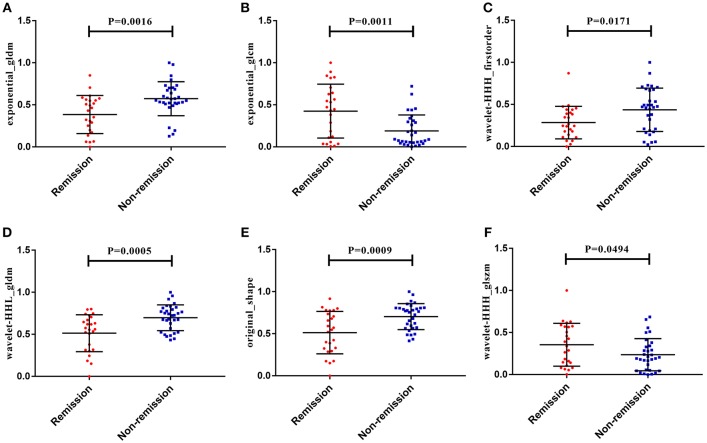
The six radiomics features showed significant differences between the different radiotherapeutic response (remission and non-remission) groups. **(A)** Exponential_gldm_DependenceVariance. **(B)** Exponential_glcm_SumEntropy. **(C)** Wavelet-HHH_firstorder_Kurtosis. **(D)** Wavelet-HHL_gldm_DependenceVariance. **(E)** Original_shape_Maximum3DDiameter. **(F)** Wavelet-HHH_glszm_SmallAreaLowGrayLevelEmphasis.

**Figure 4:**
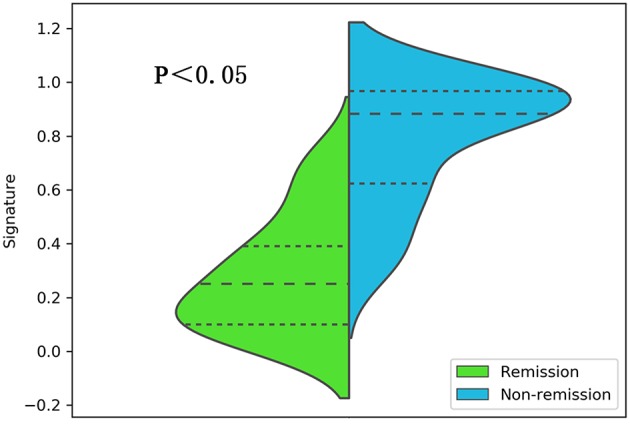
A violin plot comparing the distribution of the radiomics signatures between the different radiotherapeutic response (remission and non-remission) groups.

After making comparisons, it was found that the six selected features values were significantly different between the remission group and non-remission group (*P* = 0.0005–0.0494, Figure 3). After constructing the radiomics signature with the SVM model, a violin plot showed significant signature distribution differences between the two groups (*p* < 0.05, Figure 4). Moreover, the radiomics signature performed well in categorizing the remission and non-remission acromegaly patients after radiotherapy, reaching an AUC of 0.92 and an ACC of 0.842 (Figure 2B), and optimized sensitivity of 0.812 and specificity of 0.88. The results demonstrated that the radiomics signature could successfully predict response to radiotherapy in patients with acromegaly.

### Development, Performance, and Validation of the Radiomics Model

**Figure 5:**
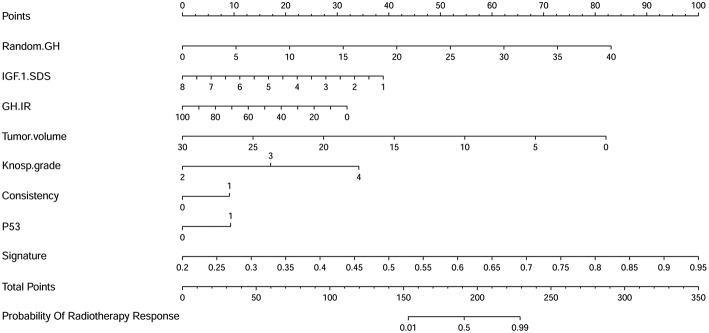
A nomogram derived from the radiomics model. This nomogram is used based on the value of the patient's eight risk factors, including radiomics signature, random GH, IGF-1 standard deviation score (SDS), GH inhibition ratio (IR), tumor volume, Knosp grade, tumor consistency, and P53 value. As shown in our previous research (49), draw a vertical line from the corresponding axis of each factor until it reaches the first “Points” line. Next, summarize the points of all risk factors, then draw a vertical line that falls vertically from the “Total Points” axis until it reaches the last axis to determine the radiotherapeutic response.

Multivariable logistic regression was used to combine the above-mentioned clinical model and radiomics signature to construct a radiomics model. The model incorporating these independent clinical and radiomics features is presented as a nomogram in Figure 5. With this new radiomics model the AUC improved to 0.96 (Figure 2B), with the DeLong test showing that its predictive efficacy was significantly higher than that of the clinical model (*P* = 0.04), although it was not significantly better than that of the radiomics signature. The optimized ACC of the radiomics model was 0.912, and its sensitivity and specificity were 0.906 and 0.920, respectively (Table 2).

**Figure 6:**
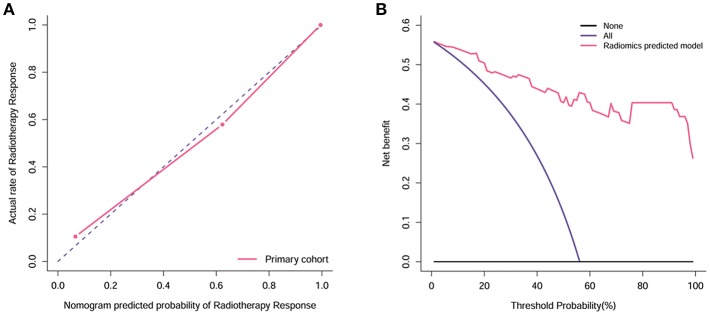
Calibration and decision curve analysis of the radiomics model. **(A)** The Y-axis represents the actual rate. The X-axis represents the predicted probability. The diagonal violet line represents a perfect prediction by an ideal model. The pink line represents the performance of the radiomics model, which shows a closer fit to the diagonal violet line representing a better prediction. (**B**) The Y-axis measures the net benefit. The pink line represents the radiomics model. The violet line represents the assumption that all patients showed remission. The black line represents the assumption that no patients showed remission.

The calibration curve demonstrated good agreement between the ground truth and predicted probabilities (*p* = 0.39, Figure 6A), with the Hosmer-Lemeshow test showing no statistical significance, indicating no significant departure from a perfect fit. Good discrimination and good calibration were therefore observed with the radiomics model.

### Clinical Usefulness

Decision curve analysis for the radiomics model is shown in Figure 6B. The radiomics model offered a net benefit in the prediction of response to radiotherapy at a threshold probability > 0.56%, thereby indicating that the radiomics model is clinically useful. The decision curve showed that this radiomics model had a relatively good performance in terms of clinical application.

## Discussion

The management of acromegaly and its complications is complex and requires a comprehensive approach coordinated by specialists in the treatment of pituitary tumors (32). For patients with acromegaly whose symptoms have not been alleviated by surgery, radiotherapy provides an alternative treatment option for controlling the disease (6, 14). Nevertheless, two studies with average follow-up times of 16.5 and 13 years found that patients with acromegaly who underwent radiotherapy had poorer metabolic status and increased mortality than patients who did not undergo radiotherapy (4, 5). Therefore, it is necessary to choose the most appropriate radiotherapy-sensitive patients for radiotherapy. However, to the best of our knowledge, no previous study has investigated a pre-radiotherapy prediction model for determining radiotherapeutic response in patients with acromegaly.

Previous studies have investigated factors associated with the efficacy of radiotherapy for acromegaly, but their results are different and contradictory. Some research has shown that smaller tumors and a higher radiation dose may result in a better radiotherapeutic response (33, 34), while Lee and colleagues found that higher radiation dose and IGF-1 levels could affect the radiotherapeutic response in acromegaly patients, but that tumor volume did not (35). However, many studies have shown that radiation dose and tumor volume did not affect the remission rate of acromegaly patients after radiotherapy (36–38), but that pre-radiotherapy GH and IGF-1 levels did show significant correlations with radiotherapeutic response (36, 37, 39). Moreover, the prognosis and treatment response should not be determined by only a single feature, with it being recognized that a combined analysis with multiple factors has more value, and may be powerful enough to change the clinical management (40, 41).

Radiomics allows high-throughput mining of quantitative imaging features from general medical images, followed by automated analysis to assist clinical decision-making (18, 25). The main steps of radiomics analysis include image collection and reconstruction, segmentation of the ROI, feature extraction and quantification, and establishment of the predictive and/or prognostic models. Typically, the quantitative features can be automatically collected be extracted from the ROI through the high-throughput technique, so as to explore the relationship with the valuable information and establish the models based on machine learning. Several recent studies have shown that radiomics has prospects in a broad array of applications, including early screening, accurate diagnosis, grading and staging, treatment and prognosis, and determination of molecular characteristics of brain tumors (42, 43). Radiomics has been shown to be important in predicting and assessing radiotherapeutic response in a variety of tumors, and its performance is significantly better than conventional methods (44), including lung cancer (45, 46), prostate cancer (47), rectal cancer (48). Therefore, we aimed to use a radiomics approach to predict the response of acromegaly to radiotherapy before treatment.

Thus, we first constructed a clinical model consisting of the seven selected important discriminatory factors. The clinical model had better prediction performance than any single clinical feature. Second, three wavelet features, two texture features, and one shape and size feature were selected as significant factors to build a radiomics signature. This could successfully categorize different responses to radiotherapy in patients with acromegaly. Finally, the radiomics model incorporating the clinical model and radiomics signature was constructed, and this showed favorable calibration and discrimination. This model was also convenient and accurate for clinical use in the pre-radiotherapy prediction of radiotherapeutic response.

To the best of our knowledge, this is the first study to establish a radiotherapeutic response predictive model. However, although our study provides significant and promising results, there are still some limitations. First, this is a single center study, and the model may behave differently on multicenter datasets with different parameters. Second, because of the limited number of patients, we adopted an LOOCV procedure to select radiomics features, instead of using independent training and test sets. Future work would benefit from training with a larger multicenter data set. Third, from clinical point of view, not only the remission rates but also the efficiency (the decline of GH and IGF-1 levels) after radiotherapy is important, patients with a significant decline to the subnormal levels can be mostly controlled with additional drug therapy, so research on patients with significantly reduced hormone levels but not reaching remission criteria is our future direction. Fourth, different subtypes of GH-producing adenomas may have different sensitivities to radiotherapy, so the related research of GH-producing adenoma subtypes is also our future research direction. Finally, radiotherapy for acromegaly is slow-acting and takes several years to play its full effect, while our patients were only followed-up for 3 years in this study, which may not fully reveal the effectiveness of radiotherapy. Therefore, more patients with longer follow-up times and complete data need to be included in future studies.

## Conclusion

In conclusion, we demonstrated that incorporating an MRI-based radiomics signature and clinical features into a radiomics model improved the accuracy of radiotherapeutic response prediction in patients with acromegaly. The radiomics model showed higher performance than any single feature, and provided an effective non-invasive tool for radiotherapeutic response prediction, which could be of assistance in deciding on individual treatment strategies for patients with acromegaly.

## Data Availability

The data used to support the findings of this study are available from the corresponding author upon request.

## Ethics Statement

This study was carried out in accordance with the recommendations of the Ethical Review Committee of Peking Union Medical College Hospital with written informed consent from all patients. The protocol was approved by the Institutional Review Board of Peking Union Medical College Hospital.

## Author Contributions

YF and SJ revised the manuscript for important intellectual content. RW and MF take final responsibility for this article. All authors provided contributions to study conception and design, acquisition of data or analysis and interpretation of data, drafting of the article, or revising it critically for important intellectual content, and final approval of the version to be published. All authors analyzed and interpreted the data.

### Conflict of Interest Statement

The authors declare that the research was conducted in the absence of any commercial or financial relationships that could be construed as a potential conflict of interest.
